# Contribution of acute-phase reaction proteins to the diagnosis and treatment of 2019 novel coronavirus disease (COVID-19)

**DOI:** 10.1017/S095026882000165X

**Published:** 2020-07-27

**Authors:** Lu Li, Changzheng Chen

**Affiliations:** Department of Ophthalmology, Renmin Hospital of Wuhan University, 238 Jiefang Road, Wuhan, Hubei Province, 430060, People's Republic of China

**Keywords:** Acute phase reaction protein, C-reactive protein, complement, COVID-19, prealbumin, serum amyloid A

## Abstract

The emergence of 2019 novel coronavirus disease (COVID-19) is currently a global concern. In this study, our goal was to explore the changing expression levels of acute-phase reaction proteins (APRPs) in the serum of COVID-19 patients and to elucidate the immunological characteristics of COVID-19. In the study design, we recruited 72 COVID-19 patients, including 22 cases of mild degree, 38 cases of moderate degree and 12 cases of severe degree. We also recruited 20 patients with community-acquired pneumonia (CAP) and 20 normal control subjects as a comparison. Fasting venous blood was taken to detect the content of complement 3 (C3), complement 4 (C4), C-reactive protein (CRP), serum amyloid A (SAA) and prealbumin (PA). When compared the COVID-19 group with the CAP and normal control groups, respectively, the mean value of CRP and SAA in the COVID-19 group (including mild, moderate and severe patients) had increased significantly (*P* < 0.01), whereas the mean values of C3, C4 and PA decreased (*P* < 0.01). For the asymptomatic or mild symptomatic patients with COVID-19, the actual aggravation of disease may be more advanced than the clinical appearances. Meanwhile, the statistical analyses indicated that the development of COVID-19 brought about a significant increase in the content of CRP and SAA (*P* < 0.01), and a decline in the content of C3, C4 and PA (*P* < 0.01). These findings suggested that the changes in the level of APRPs could be used as indicators to identify the degree and progression of COVID-19, and the significant changes might demonstrate the aggravation of disease. This study provided a new approach to improve the clinical management plan and prognosis of COVID-19.

## Introduction

The 2019 novel coronavirus disease (COVID-19) is a highly infectious pneumonia caused by severe acute respiratory syndrome coronavirus 2 (SARS-CoV-2) [1, 2]. It was first reported in Wuhan, the Hubei province of China and then broke out in several places, spreading across the country. As of 2 April 2020, 950,000 people of 204 countries had been infected with COVID-19. Based on the confirmed cases in clinics, several studies have reported the characteristics of patients with COVID-19 with respect to epidemiology, clinical symptoms, laboratory examination, imaging examination, treatments and clinical outcome [1–3].

Coronaviruses have a capsule membrane and a linear single-stranded RNA genome. They are classified into four genera (*α*, *β*, *γ* and *δ*). The SARS-CoV-2 belongs to the same genus as SARS-CoV (severe acute respiratory syndrome-coronavirus) and MERS-CoV (Middle East respiratory syndrome-coronavirus), however, the genetic sequence similarity of SARS-CoV-2 to SARS-CoV and MERS-CoV is about 70% and 40%, respectively [4]. The previous studies have shown that the receptor protein of SARS-CoV-2 is angiotensin-converting enzyme 2 (ACE2), which is highly homologous to that of SARS-CoV [5]. Additionally, the computer structure simulation predicted that the receptor protein of SARS-CoV-2 is ACE2, which is the same as SARS-CoV [6]. Furthermore, in vitro, the cell experiments also proved that ACE2 was the receptor protein of SARS-CoV-2 [7].

The human immune system plays a key role in the defence against pathogenic micro-organisms, and the strength of the immune system determines the disease status and prognosis [8]. Acute-phase reaction proteins (APRPs) are reactive proteins, which are directly manifested as part of the body's stress response. In the case of infection or tissue damage, the inflammatory response stimulates the liver to synthesise and release APRPs in large quantities − a reaction that has been proved to be of great significance in suppressing inflammation and regulating immunity in the body [9]. Currently, there are no systematic analyses regarding the immunological characteristics of COVID-19. In this paper, we evaluated the immune response to SARS-CoV-2 by comparing the levels of some APRPs in the serum among the COVID-19 patients, community-acquired pneumonia (CAP) patients and normal people. We hope these findings can provide references for the effective treatment and control of COVID-19.

## Materials and methods

### Study design and participants

This is a respective and single-centre study that involved 72 COVID-19 patients, 20 age-matched CAP patients and 20 age-matched normal adults. This study was approved by the Ethics Committee of Renmin Hospital of Wuhan University, and followed the tenets set forth in the Declaration of Helsinki. Written informed consent was obtained from all participants involved.

COVID-19 groups: we recruited 72 COVID-19 patients, including 39 males and 33 females, from 27 January to 28 March, 2020, at Renmin Hospital of Wuhan University, Wuhan, China. The general clinical data, which includes age, clinical symptoms, previous medical history and complications for all patients, were collected. The criteria for enrolment and exclusion: the patients who were confirmed positive based on the results of nucleic acid test for novel coronavirus from nasopharyngeal swabs were enrolled, and the patients who had previous treatment for COVID-19 were excluded. According to the national guideline of COVID-19 in China, the patients were classified into three subgroups – mild, moderate and severe group [10]. In the mild group, there were 22 patients (12 males and 10 females). They had no clinical or only mild clinical symptoms, and chest CT examinations were normal without manifestation of viral pneumonia. In the moderate group, there were 38 patients (20 males and 18 females). For the patients in this group, the medical interventions were needed to relieve the fever and respiratory symptoms. And the chest CT examinations showed the manifestation of viral pneumonia. In the severe group, based on the criteria of the national guideline of COVID-19 in China, 12 patients (7 males and 5 females) were selected. The patients in this group had one or more of the following characteristics: (1) respiratory distress (≥ 30 breaths/min); (2) oxygen saturation ≤ 93% at rest; (3) arterial partial pressure of oxygen (PaO_2_)/fraction of inspired oxygen (FiO_2_) ≤ 300 mmHg (1 mmHg = 0.133 kPa). The levels of APRPs were measured from the fasting venous blood taken in the morning within 3 days before medical intervention.

CAP groups: we selected 20 age-matched CAP patients (10 males and 10 females), who were admitted to the same hospital from January to March of 2019. The patients in this group had simple CAP without any other diseases, and the pathogenic viruses included influenza A virus (six patients), influenza B virus (ten patients) and adenovirus (four patients). Based on the patients' record, the contents of APRPs were obtained from the fasting venous blood taken in the morning within 3 days before medical intervention.

Normal group: we selected 20 age-matched healthy adults (10 males and 10 females), who were given health check in the physical examination centre of the same hospital from January to March of 2019. All the enrolled participants in this group were in good health with no history of local and systemic diseases, and no discomfort during the physical examination. The results of heath check showed no obvious abnormalities. The levels of APRPs were measured from the fasting venous blood taken in the morning.

### Laboratory inspection

All the blood samples were analysed in the laboratory of clinical biochemistry, Renmin Hospital of Wuhan University. The targeted APRPs included complement 3 (C3), complement 4 (C4), C-reactive protein (CRP), serum amyloid A (SAA) and prealbumin (PA). The levels of CRP and PA were determined by the Hitachi 7170 automatic biochemical analyser from Japan. The results of C3 and C4 were obtained via the automatic immune analyser IMMAGE800 (Beckman, USA). And the content of SAA was measured by the SAA quantitative kit from Shanghai Upper Biomedical Company. All the operation procedures followed the manufacturers' instructions.

### Statistical analysis

We used Prism 8 (GraphPad Software, Inc., La Jolla, CA, USA) for the statistical analysis. The continuous measurements were presented as mean and standard deviation (SD). The counting data were expressed by rate (%). The independent sample *T*-test was used to detect the data between two groups. The *P* value < 0.05 indicated a statistically significant difference.

## Results

The ages of patients in COVID-19 group ranged from 22 to 71 years, with an average of 45.9 ± 14.3 years. In the mild COVID-19 group, their ages ranged from 22 to 61 (39.1 ± 12.2) years, in the moderate COVID-19 group, their ages ranged from 24 to 71 (47.8 ± 14.4) years and in the severe COVID-19 group, their ages ranged from 28 to 71 (52.1 ± 14.2) years. The ages of patients in CAP group ranged from 29 to 68 years, with an average of 55.0 ± 13.8 years, and the ages of participants in normal group ranged from 29 to 68 years, with an average of 51.2 ± 11.1 years.

The statistical characteristics of the content of the target proteins, C3, C4, CRP, SAA and PA, in each group are shown in Table 1.

**Table 1.** Number, percent of abnormality (Abn.) and mean value of APRPs in each group

Group	C3		C4		CRP		SAA		PA	
	Abn. *N* (%)	Mean (0.81–1.6) (g/l)	Abn. *N* (%)	Mean (0.1–0.4) (g/l)	Abn. *N* (%)	Mean (0–10) (mg/l)	Abn. *N* (%)	Mean (0–10) (mg/l)	Abn. *N* (%)	Mean (250–400) (mg/l)
COVID-19 (*n* = 72)	43 (60)	0.77 ± 0.27	16 (22)	0.20 ± 0.11	63 (88)	20.38 ± 10.50	72 (100)	151.53 ± 71.20	61 (85)	170.15 ± 61.79
Mild COVID-19 (*n* = 22)	8 (36)	0.92 ± 0.20	4 (18)	0.31 ± 0.12	13 (59)	11.08 ± 2.44	22 (100)	96.53 ± 31.00	15 (68)	214.78 ± 48.80
Moderate COVID-19 (*n* = 38)	23 (61)	0.77 ± 0.28	4 (11)	0.18 ± 0.17	38(100)	20.45 ± 7.40	38 (100)	148.94 ± 54.58	34 (89)	163.87 ± 56.53
Severe COVID-19 (*n* = 12)	12(100)	0.51 ± 0.12	8 (67)	0.10 ± 0.03	12(100)	37.25 ± 5.72	12 (100)	260.58 ± 42.67	12 (100)	108.20 ± 32.23
CAP (*n* = 20)	7 (35)	1.28 ± 0.41	8 (40)	0.32 ± 0.17	3 (15)	7.14 ± 2.61	18 (90)	75.59 ± 63.54	13 (65)	234.95 ± 29.82
Normal (*n* = 20)	0 (0)	1.16 ± 0.13	0 (0)	0.29 ± 0.05	0 (0)	0.40 ± 0.28	0 (0)	3.94 ± 0.87	0 (0)	325.30 ± 30.48

**Fig. 1.**
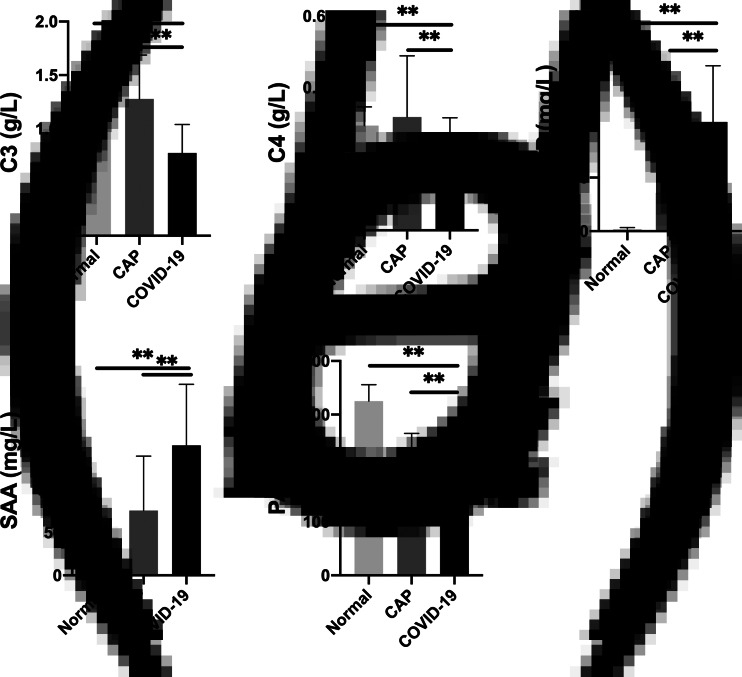
Different APRP contents in COVID-19 patients, CAP patients and the normal control group (**P < 0.01).

Compared with the results of CAP and normal control groups, the mean values of C3, C4, CRP, SAA and PA in the COVID-19 group (including mild, moderate and severe patients) showed significant changes (*P* < 0.01) (Fig. 1). The mean values of CRP and SAA increased significantly, whereas the mean values of C3, C4 and PA decreased. Meanwhile, the mean value of C4 (0.20 ± 0.11 g/l) was still within the normal reference range (0.1–0.4 g/l).

**Fig. 2.**
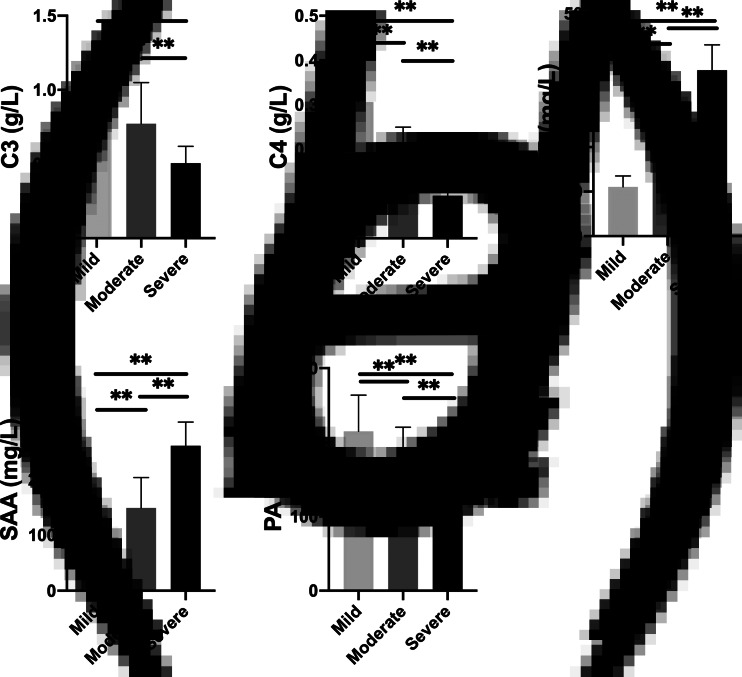
Different APRP contents in mild, moderate and severe cases of COVID-19 (*P < 0.05, **P < 0.01).

In the COVID-19 group, the content of APRPs varied greatly among the mild, moderate and severe patients (Fig. 2). The aggravation of the disease resulted in a significant increase of CRP and SAA content (*P* < 0.01), accompanied by a decrease of C3, C4 and PA content (*P* < 0.01).

In the group of 22 mild COVID-19 patients, the contents of C3 were in the normal reference range (0.81–1.6 g/l) in 14 patients (64%), and the contents of C4 were in the normal reference range (0.1–0.4 g/l) in 18 patients (82%). There were eight patients (36%) in which case the content of C3 was lower, but the content of C4 was normal. In four patients (18%), the content of C4 was slightly higher (no more than 0.542 g/l). The mean value of C3 and C4 were in the normal reference range. Additionally, in mild COVID-19 patients, there were nine patients (41%) that had normal CRP content whereas SAA increased significantly above the normal range. The mean value of the content of CRP (11.08 ± 2.44 mg/l) was near the critical value (10 mg/l), and the mean value of the content of SAA exceeded the normal range significantly (*P* < 0.01). In the mild COVID-19 group, seven patients (32%) had normal levels of PA (250–400 mg/l).

In the group of 38 moderate COVID-19 patients, 15 patients (39%) had normal levels of C3, and 34 patients (89%) had normal levels of C4. There were 19 patients (50%) who had lower C3 contents but normal C4 contents. Moreover, four moderate COVID-19 patients (11%) had lower contents of both C3 and C4 below the normal range, although the mean value of C4 was still in the normal reference range. In the moderate COVID-19 group, all patients had significantly higher levels of CRP and SAA. There were four patients (11%) who had normal PA content, but no more than 287.97 mg/l.

In the group of 12 severe COVID-19 patients, all patients had lower levels of C3, and eight of them (67%) also had lower levels of C4 than the normal reference range. The mean value of C4 (0.10 ± 0.03 g/l) reached the critical value (0.1 g/l). All patients had significantly higher levels of CRP and SAA, and lower levels of PA. The minimum value of PA in this group was 65.35 mg/l, which was reduced by 80% compared with the mean value of PA in the normal control group (325.30 ± 30.48 mg/l).

**Fig. 3.**
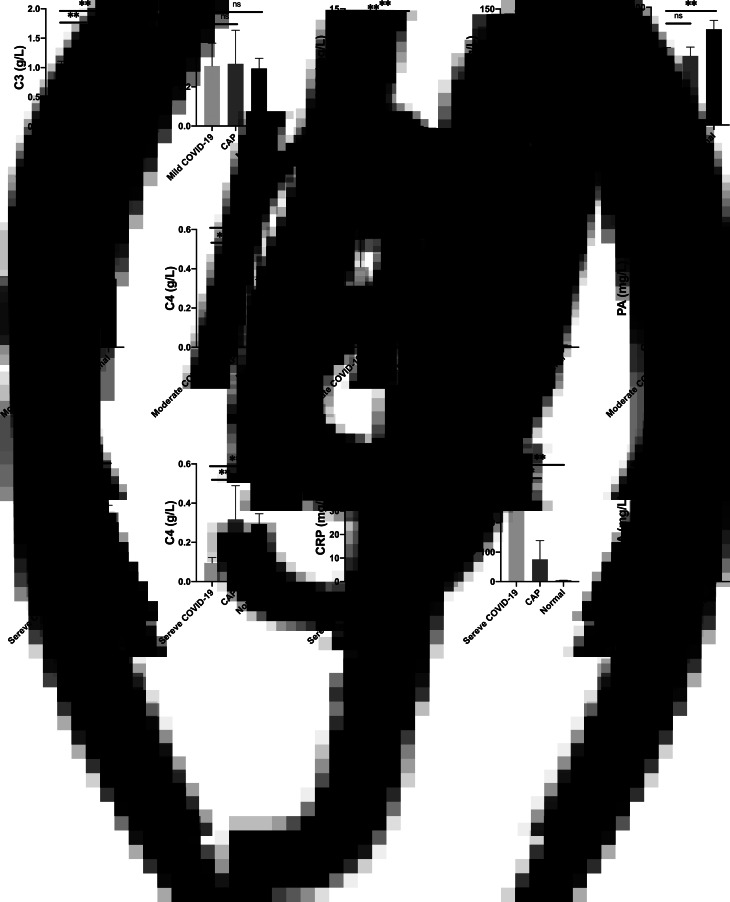
Different APRP contents in mild, moderate and severe cases of COVID-19, CAP patients and the normal control group (ns *P* > 0.05, ** *P* < 0.01).

Compared with the patients in CAP group, the patients in mild COVID-19 group showed no significant difference in the content of C4, SAA and PA, however, the mild COVID-19 patients complained less about discomfort (Fig. 3).

## Discussion

As a new infectious disease, there are still many mysteries surrounding COVID-19 that remain to be solved. A newly discovered coronavirus, SARS-CoV-2 has many similarities with SARS-CoV and MERS-CoV, although the pathogenic mechanisms and immune responses may not be identical.

On the basis of our observation, for the participants in the normal control group, the levels of APRPs were all within the normal range. In the mild COVID-19 group, some patients had normal content of C4, CRP and PA, but lower content of C3 and higher content of SAA. In the moderate and severe groups, the APRPs values of the patients changed further with the trend (i.e. CRP and SAA increased, and C3, C4 and PA decreased). These changes in the severe group were greater than that of the moderate group. These findings suggested that the significant changes in the levels of APRPs signalled an aggravation of COVID-19, and the markers of C3 and SAA were more sensitive than C4 and CRP. Additionally, the degree and development of COVID-19 might be identified by the changes in content of APRPs, which means that the prognosis and clinical management plan could be improved accordingly.

On the one hand, compared with the CAP group, the patients in the mild COVID-19 group exhibited a similar change in the levels of APRPs, but complained less about the discomfort. On the other, for the patients with similar degrees of clinical symptoms, we found that the changes of APRPs in COVID-19 group were more significant than that in the CAP group. For the asymptomatic or mild symptomatic patients with COVID-19, the actual aggravation of disease may be more advanced than the clinical appearances. In this case, the levels of APRPs can be considered as markers to identify the development of COVID-19 and improve the prognosis.

The complement system is an important immune surveillance system, functioning as an innate barrier of the body and regulating adaptive immunity against infection and inflammation [11]. It has three activation pathways: the classical pathway, the alternate pathway and the mannan-binding lectin pathway [11]. As the most two abundant proteins in complement system, C3 plays key roles through all three activation pathways and C4 functions mainly through the classical pathway [11]. In this study, we found that both the content of C3 and C4 declined as the development of COVID-19, however, the decrease of C3 occurred earlier than C4. We speculated that infection with SARS-CoV-2 somehow activated the alternate pathway in the complement system first, and thus consumed a large amount of C3. However, the C4, which is activated by the classical pathway, was consumed less because the body produced little antigen−antibody immune complex in the early or mild stage of COVID-19.

Both CRP and SAA can respond immediately after infection or tissue damage. CRP is responsible for the complement activation and opsonisation, the modulation of monocytes and macrophages, the production of cytokine and the prevention of tissue migration of neutrophils [9]. SAA is associated with the inhibitory effects on fever, the oxidative burst of neutrophilic granulocytes, immune response, platelet activation and the chemotactic effect on monocytes [9]. In our study, SAA was more sensitive than CRP. For some patients in the mild COVID-19 group, the value of CRP was still normal, but the value of SAA increased significantly. These results were consistent with the conclusion that CRP was more sensitive in bacterial infections than in viral infections, whereas, SAA increased more quickly and significantly than CRP in viral infections [9].

PA is synthesised in liver cells and secreted by pancreatic islet cells. It is a plasma transporter protein with a 12 h half-life. It is a negative APRP and reduced rapidly in response to acute infection. The changes in the level of PA is regarded as one of the specific indexes of liver function damage and a sensitive indicator to reflect low protein status. For the patients with COVID-19, the significant decrease of PA may be due to (1) its consumption as a non-specific host defence substance in the elimination of the toxic metabolites after infection; or (2) its short half-life and reduced synthesis.

There were some limitations to this study. (1) The systemic diseases, such as cardiovascular and endocrine diseases, were not excluded and may have influenced the expression level of APRPs. (2) For the patients in the COVID-19 and CAP groups, we were unable to assess the premorbid immune state. (3) Due to the limited information on patients' records, the patients in CAP group were not classified based on the severity of disease; (4) Since January 2020, the Renmin Hospital of Wuhan University has been converted into a designated hospital for COVID-19 patient amid coronavirus outbreak. Therefore, for the participants in the CAP and normal control groups, we could only gather the information from the database of our hospital rather than multiple resources. All of these factors may bias the statistical results to some extent. To reduce the effects of season and temperature on hormone levels, we selected the patients who were hospitalised in the same season of 2019.

The COVID-19 patients in ICU were not included in this study. However, based on the current trend, the changes in content of APRPs may be more significant in ICU patients, which means that the levels of C3, C4 and PA may be even lower, and the levels of SAA and CRP may be even higher. Additionally, we only studied the APRPs in the acute stage of COVID-19, however, the immunological characteristics in convalescence are equally important for better understanding and management of this infectious disease. For these reasons, further studies are necessary to investigate more effective treatment options.

## Data Availability

The data that support the findings of this study are available by request to the corresponding author.
